# Genetically proxied PCSK9 inhibition is associated with reduced psoriatic arthritis risk

**DOI:** 10.1007/s00011-024-01850-3

**Published:** 2024-02-11

**Authors:** Junhong Li, Jianfeng Li, Chengkai Lin, Jiaxiang Zhou, Jianmin Wang, Fuan Wang, Haizhen Li, Zhiyu Zhou

**Affiliations:** 1 Innovation Platform of Regeneration and Repair of Spinal Cord and Nerve Injury, Guangming District, The Seventh Affiliated Hospital, Sun Yat-Sen University, 66 Gongchang Road, Shenzhen, 518107 China; 2 Department of Orthopaedics and Trauma, The Affiliated Hospital of Yunnan University, Yunnan University, Kunming, 650091 China; 3 Department of Orthopaedic Surgery, The Affiliated Hospital of Qingdao University, Qingdao, 266003 China; 4 Department of Spinal Surgery, Yantaishan Hospital, Yantai, 264003 China; 5 Guangdong Provincial Key Laboratory of Orthopaedics and Traumatology, the First Affiliated Hospital of Sun Yat-Sen University, Guangzhou, 510080 China

**Keywords:** Lipid-lowering drugs, PCSK9, Psoriatic arthritis, Therapeutic target, Mendelian Randomization

## Abstract

**Background:**

Lipid pathways play a crucial role in psoriatic arthritis development, and some lipid-lowering drugs are believed to have therapeutic benefits due to their anti-inflammatory properties. Traditional observational studies face issues with confounding factors, complicating the interpretation of causality. This study seeks to determine the genetic link between these medications and the risk of psoriatic arthritis.

**Methods:**

This drug target study utilized the Mendelian randomization strategy. We harnessed high-quality data from population-level genome-wide association studies sourced from the UK Biobank and FinnGen databases. The inverse variance-weighted method, complemented by robust pleiotropy methods, was employed. We examined the causal relationships between three lipid-lowering agents and psoriatic arthritis to unveil the underlying mechanisms.

**Results:**

A significant association was observed between genetically represented proprotein convertase subtilisin/kexin type 9 (PCSK9) inhibition and a decreased risk of psoriatic arthritis (odds ratio [OR]: 0.51; 95% CI 0.14–0.88; *P* < 0.01). This association was further corroborated in an independent dataset (OR 0.60; 95% CI 0.25–0.94; *P* = 0.03). Sensitivity analyses affirmed the absence of statistical evidence for pleiotropic or genetic confounding biases. However, no substantial associations were identified for either 3-hydroxy-3-methylglutaryl-CoA reductase inhibitors or Niemann–Pick C1-like 1 inhibitors.

**Conclusions:**

This Mendelian randomization analysis underscores the pivotal role of PCSK9 in the etiology of psoriatic arthritis. Inhibition of PCSK9 is associated with reduced psoriatic arthritis risk, highlighting the potential therapeutic benefits of existing PCSK9 inhibitors.

## Introduction

Psoriatic arthritis (PsA) is a chronic inflammatory disorder that primarily targets joints and various components of the musculoskeletal system [1]. Recent studies indicate that psoriatic arthritis, affecting up to 30% of individuals with psoriasis, has a prevalence of 6–25 cases per 10,000 people in the USA [2]. The clinical presentations of PsA are diverse, encompassing peripheral joint inflammation, inflammatory back discomfort, enthesitis, and tenosynovitis [3]. The debilitating effects of PsA are now recognized to be on par with other inflammatory arthritic conditions such as rheumatoid arthritis (RA) and axial spondyloarthritides (axSpA) [2]. The functional impairments stemming from PsA often result in decreased work efficiency, increased absenteeism, and a marked decline in the quality of life for those affected [4].

A significant observation among PsA patients is the prevalence of disrupted lipid metabolism, which elevates the risk of cardiovascular complications [5–7]. Thus, screening for dyslipidemia becomes imperative for those diagnosed with PsA [8, 9]. While lipid-lowering medications are pivotal in managing cardiovascular risks [10], their direct impact on PsA treatment remains a topic of ongoing research. The efficacy of several mainstream lipid-lowering drugs in treating PsA symptoms is yet to be conclusively established.

Exploring lipid pathway interventions in PsA is compelling due to several factors. Some lipid-lowering medications exhibit anti-inflammatory properties, suggesting their potential as therapeutic agents for PsA. The unclear pathogenesis of PsA highlights the importance of identifying lipid-related causal pathways. Using lipid-lowering drugs that target both lipid imbalances and PsA offers personalized treatment options, especially for those with a strong family history and dyslipidemia. If these drugs show disease-modifying effects, they could be repurposed for PsA treatment, reducing immunosuppression risks. However, traditional pharmacoepidemiologic designs present challenges in obtaining solid evidence.

The inherent genetic variations related to protein drug targets can shed light on potential clinical outcomes [11]. Leveraging genetic instrumental variable analyses, or Mendelian randomization (MR), offers a quasi-randomized approach, providing a more resilient framework against biases typically seen in conventional epidemiological studies [12]. Our study's objective was to employ a two-sample MR to delve into the relationship between PsA risk and three genetically indicated lipid-lowering drugs: proprotein convertase subtilisin/kexin type 9 (PCSK9) inhibitors (eg, alirocumab), 3-hydroxy-3-methylglutaryl-CoA reductase (HMGCR) inhibitors (ie, statins), and Niemann–Pick C1-like 1 (NPC1L1) inhibitors (i.e., ezetimibe).

## Materials and methods

**Fig. 1:**
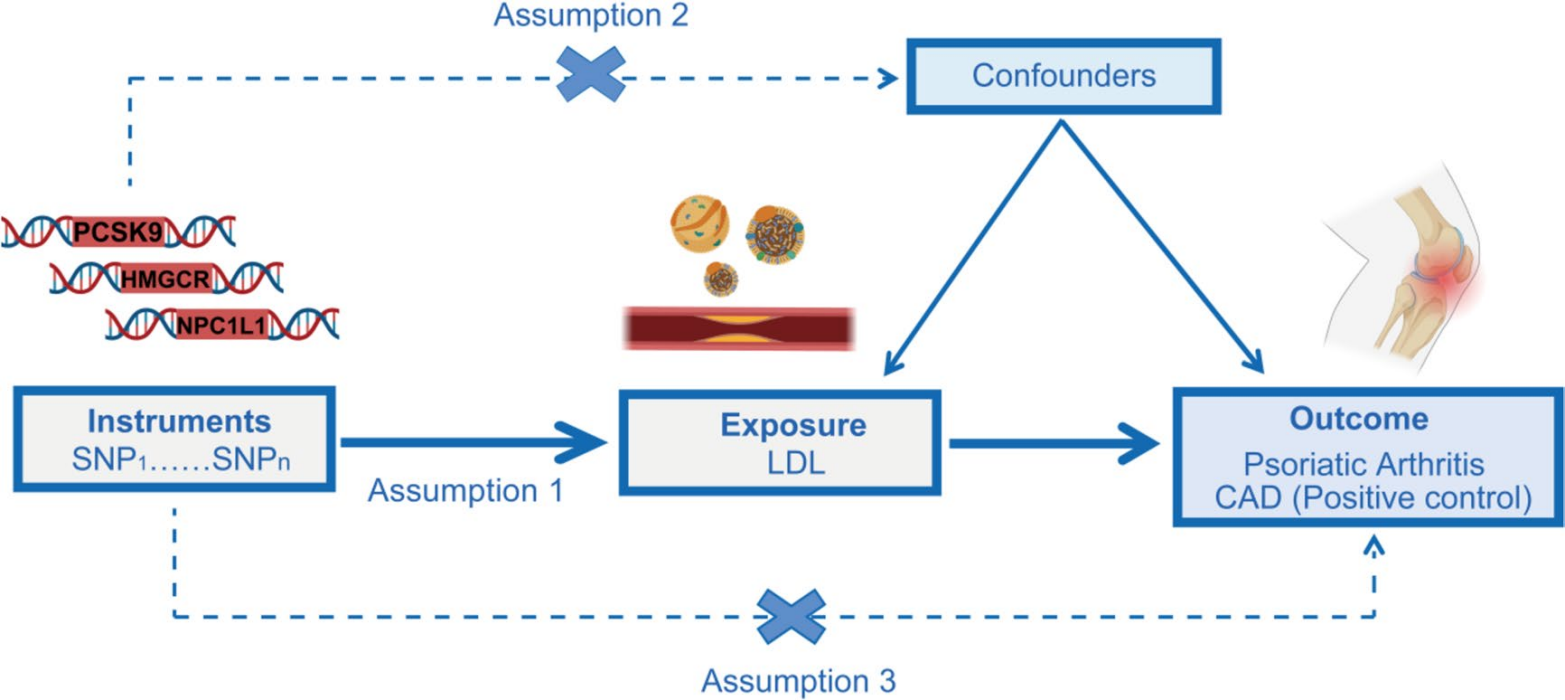
Research overview and design of drug target Mendelian randomization analysis. To establish a causal link, the following criteria must be met: (1) instrumental variables should be independent of confounders (indicated by dashed lines), (2) instrumental variables must be associated with the exposure (depicted by solid lines), and (3) instrumental variables shouldn't have a direct connection to the outcome (represented by dashed lines). PCSK9, proprotein convertase subtilisin/kexin type 9; HMGCR, 3-hydroxy-3-methylglutaryl CoA reductase; NPC1L1, Niemann-Pick C1-like 1; LDL, low-density lipoprotein

This research employed deidentified summary data derived from prior genome-wide association studies (GWAS). Ethical clearances were secured for all original investigations, and relevant references are comprehensively documented. The study design is illustrated in Fig. 1.

### Genetic proxies for lipid-lowering drugs

We selected low-density lipoprotein (LDL) as a biomarker due to the demonstrated efficacy of three lipid-lowering drugs in reducing LDL cholesterol levels. To the best of our knowledge, we utilized the most comprehensive GWAS meta-analysis currently available, which has established genetic associations with LDL, covering 12,321,875 SNPs with a sample size of 440,546 [13].

We identified variants associated with LDL at a genome-wide significance level (*P* < 5 × 10^−8^). These variants exhibited minimal correlation, with a linkage disequilibrium threshold of *r*^2^ < 0.3, as determined using PLINK and referencing phase 3 version 5 of the 1000 Genomes Project. We focused on regions within ± 100 kb of the PCSK9 gene (build GRCh37/hg19: chromosome 1: 55,505,221, 55,530,525) for PCSK9 inhibitors (e.g., alirocumab or evolocumab), the HMGCR gene (chromosome 5: 74,632,154, 74,657,929) as an instrumental variable for statins, and the NPC1L1 gene (chromosome 7: 44,552,134, 44,580,914) as an instrumental variable for ezetimibe.

Considering the observed correlation between lipids and PsA, we explored the potential association between genetically predicted LDL levels and the risk of PsA. To instrument LDL, we utilized genome-wide significant variants with pairwise correlations below r^2^ < 0.001, excluding variants from the three previously mentioned drug target gene regions.

### Genetic association for psoriatic arthritis

We obtained genetic associations for PsA from the GWAS data with the largest number of SNPs in the FinnGen database (eTable 1). This dataset has a sample size of 213,879, which includes 16,380,462 SNPs, 1637 patients, and 212,242 controls. PsA is defined according to the standards in the International Statistical Classification of Diseases and Related Health Problems, Tenth Revision (ICD-10). The ICD-10 code is L40.5.

To validate our analyses, we sourced genetic associations for PsA from an alternative dataset comprising a sample size of 218,792, encompassing 1455 patients and 217,337 controls (eTable 1).

### Statistical analysis and MR assumptions

We employed the inverse variance-weighted approach with multiplicative random effects to derive a weighted mean from individual variant estimates [14]. A valid instrumental variable adheres to three fundamental assumptions [15]. Firstly, the variants should exhibit a significant association with the intended exposure. We calculated the F statistics for drug target instruments, using the ratio of the squared β to the squared standard error. An F statistic exceeding 10 indicates a sufficiently robust instrument strength [16].

Secondly, there should be no mutual causal relationship between the variants and the outcome. To mitigate confounding due to inherent population structures, we restricted our analysis to populations of European descent.

Thirdly, the variants should influence the outcome solely through the specified risk factor. To assess the resilience of our primary inverse variance-weighted estimates against horizontal pleiotropy (where instruments influence the outcome via factors other than the exposure), we employed the MR Egger [17], weighted median [18], and weighted mode [19] methodologies. We referenced the PhenoScanner [20, 21], a meticulously curated genotype–phenotype database, to identify correlations between variants designated for each drug target and other potential traits indicative of pleiotropic pathways. Notably, traits linked to smoking [22] and psoriasis [23], which are established risk determinants for PsA disease, were scrutinized. In our sensitivity analyses, variants that exhibited associations with these and other relevant traits, surpassing the threshold of *P* < 1 × 10^−5^, were excluded to mitigate potential sources of pleiotropy.

### Supplementary analysis

To ensure the robustness of our primary analyses, we conducted a supplementary evaluation. We validated our instruments by employing coronary artery disease as a positive control outcome, considering the well-established therapeutic effects of lipid-lowering drugs in this domain. Genetic associations were sourced from a GWAS encompassing 42,096 clinically validated cases (e.g., myocardial infarction, acute coronary syndrome, chronic stable angina, or coronary stenosis exceeding 50%) [24].We also used data from another study for validation [25].

## Results

In primary analysis utilizing the PsA dataset from the FinnGen database, we identified 33 genetic variants representing LDL reduction via PCSK9 inhibition (with a mean F statistic of 167), 19 variants for HMGCR (with a mean F statistic of 135), and 6 for NPC1L1 (with a mean F statistic of 72) as detailed in Table 1.

**Table 1:** Genetic variants used to instrument each lipid-lowering drug targetSA

Exp	SNP	Chr	Pos	EA	OA	EAF	Beta	Se	P value	F
PCSK9	rs6691964	1	55,433,978	A	G	0.092472	-0.0234719	0.00358388	5.80E-11	40.74048769
PCSK9	rs556369867	1	55,491,135	T	C	0.330585	0.0175746	0.00243048	4.80E-13	60.23211898
PCSK9	rs72909541	1	55,494,301	T	C	0.046097	-0.0334061	0.00501479	2.70E-11	43.24046364
PCSK9	rs150119739	1	55,520,938	A	G	0.045318	0.0452728	0.00520209	3.20E-18	78.14487409
PCSK9	rs7525503	1	55,522,558	T	G	0.02036	0.0454642	0.0075822	2.00E-09	36.32774896
PCSK9	rs11587071	1	55,522,674	T	C	0.168881	-0.0282322	0.00279415	5.30E-24	98.59387078
PCSK9	rs10493176	1	55,538,552	G	T	0.07579	-0.0531381	0.00394676	2.60E-41	174.3354587
PCSK9	rs3976734	1	55,489,960	G	A	0.374504	-0.0297494	0.00231882	1.10E-37	182.7413453
PCSK9	rs200730299	1	55,491,853	C	A	0.195034	-0.0543492	0.00278155	5.10E-85	408.9747062
PCSK9	rs17192725	1	55,496,131	A	G	0.095408	0.0305717	0.00365832	6.40E-17	71.08305236
PCSK9	rs17111503	1	55,503,448	G	A	0.268141	0.0406795	0.00235743	1.00E-66	286.3144736
PCSK9	rs7546522	1	55,516,713	T	C	0.155442	-0.0168117	0.00295297	1.20E-08	32.6943445
PCSK9	rs2483205	1	55,518,316	T	C	0.438633	-0.0295845	0.00214514	2.90E-43	189.9691892
PCSK9	rs11583974	1	55,551,718	A	G	0.042146	0.0314531	0.00517068	1.20E-09	35.19140196
PCSK9	rs56349475	1	55,576,102	C	T	0.024601	-0.0475957	0.00671909	1.40E-12	47.90016489
PCSK9	rs79396670	1	55,588,142	A	G	0.035496	-0.0336489	0.00562029	2.10E-09	34.15687648
PCSK9	rs146273942	1	55,453,841	A	G	0.023188	-0.0538858	0.00722418	8.70E-14	57.95612818
PCSK9	rs2479420	1	55,492,190	T	C	0.73803	-0.0283879	0.0023826	9.90E-33	137.3240966
PCSK9	rs11810371	1	55,496,861	A	G	0.043743	-0.0294547	0.00507333	6.40E-09	31.97740435
PCSK9	rs11591147	1	55,505,647	T	G	0.017468	-0.348456	0.00793088	1.00E-200	1843.821775
PCSK9	rs11206513	1	55,507,649	T	C	0.600617	0.0316517	0.0021463	3.20E-49	211.8406735
PCSK9	rs11206517	1	55,526,428	G	T	0.033149	0.0680285	0.00580615	1.00E-31	130.7253793
PCSK9	rs2495517	1	55,448,842	G	A	0.794271	0.0177548	0.0025792	5.80E-12	45.38996213
PCSK9	rs12732125	1	55,470,153	T	C	0.020368	-0.10344	0.0073736	1.00E-44	188.1885252
PCSK9	rs2479395	1	55,484,582	C	T	0.668453	0.0125674	0.00221762	1.50E-08	30.84299521
PCSK9	rs77875082	1	55,485,042	A	G	0.032388	0.0481535	0.00605559	1.80E-15	64.03589791
PCSK9	rs41294821	1	55,513,183	T	C	0.022807	-0.0386615	0.00705365	4.20E-08	29.35311066
PCSK9	rs472495	1	55,521,313	T	G	0.648959	0.0425743	0.00218093	7.30E-85	364.1230994
PCSK9	rs530804537	1	55,583,210	A	G	0.011303	-0.192336	0.00997554	7.80E-83	364.5495945
PCSK9	rs55637835	1	55,466,303	T	C	0.120881	-0.0187129	0.00324835	8.40E-09	32.78985634
PCSK9	rs12739979	1	55,496,648	T	C	0.246521	-0.0202563	0.00254032	1.50E-15	67.16311122
PCSK9	rs72660548	1	55,500,978	G	C	0.018458	0.0509816	0.00777535	5.50E-11	41.49355463
PCSK9	rs45613943	1	55,518,622	C	T	0.048725	-0.0340702	0.0048672	2.60E-12	47.41036718
HMGCR	rs75240579	5	74,624,484	T	C	0.048363	-0.0372115	0.00487202	2.20E-14	56.15824795
HMGCR	rs2006760	5	74,562,029	G	C	0.205486	0.03556	0.00261075	3.00E-42	181.9725737
HMGCR	rs62366588	5	74,664,987	A	C	0.065948	-0.0271295	0.00433093	3.70E-10	39.94982959
HMGCR	rs141642272	5	74,615,209	C	G	0.026705	0.0532822	0.00653971	3.70E-16	65.02565811
HMGCR	rs55727654	5	74,651,864	A	G	0.14871	0.042154	0.0029315	6.90E-47	198.2942
HMGCR	rs111353455	5	74,623,949	A	G	0.085844	0.0243909	0.00372718	6.00E-11	41.13824406
HMGCR	rs2303152	5	74,641,707	A	G	0.101442	0.0333589	0.00345272	4.40E-22	89.39119883
HMGCR	rs116153450	5	74,729,433	A	C	0.04623	-0.0303618	0.00499242	1.20E-09	35.81600769
HMGCR	rs12916	5	74,656,539	C	T	0.400537	0.0621175	0.00212705	1.70E-187	817.8199832
HMGCR	rs17562727	5	74,682,474	C	T	0.027617	0.0394972	0.00635898	5.30E-10	36.91493872
HMGCR	rs80324692	5	74,717,761	T	C	0.081157	-0.0260509	0.00385694	1.40E-11	44.59395641
HMGCR	rs115845757	5	74,563,700	A	G	0.019	0.048608	0.00785612	6.10E-10	38.80571168
HMGCR	rs17648121	5	74,650,106	T	C	0.029877	0.0619849	0.00619367	1.40E-23	98.14125847
HMGCR	rs140092661	5	74,682,600	T	A	0.034128	0.0329927	0.00582168	1.50E-08	31.61670332
HMGCR	rs12659331	5	74,757,657	C	A	0.054326	0.0251785	0.00459392	4.20E-08	28.69831922
HMGCR	rs72633963	5	74,630,829	A	G	0.1238	0.0564278	0.00316653	4.90E-71	304.5296074
HMGCR	rs10051965	5	74,560,487	T	C	0.369925	0.0410063	0.00216518	5.40E-80	345.5945975
HMGCR	rs35122945	5	74,610,293	C	A	0.067368	-0.0281057	0.00423755	3.30E-11	43.73359482
HMGCR	rs4703665	5	74,602,898	C	T	0.848709	0.0244938	0.00297742	1.90E-16	67.88447107
NPC1L1	rs217399	7	44,592,091	T	C	0.443739	-0.0213142	0.00211012	5.50E-24	98.8236711
NPC1L1	rs73107478	7	44,596,644	C	A	0.079159	0.0259282	0.00392582	4.00E-11	43.18088467
NPC1L1	rs11763759	7	44,570,067	C	T	0.303527	-0.0133498	0.00234076	1.20E-08	33.19732916
NPC1L1	rs2073547	7	44,582,331	G	A	0.184007	0.0355498	0.00267287	2.30E-40	167.2550498
NPC1L1	rs148825701	7	44,559,803	T	C	0.212133	0.017365	0.00256025	1.20E-11	44.4092852
NPC1L1	rs12666108	7	44,586,578	C	T	0.091516	0.0249436	0.00363876	7.10E-12	45.58235923

Exp, exposure; Chr, chromosome; Pos, position; EA, effect allele; OA, other allele; EAF, effect allele frequency; Se, standard error; PCSK9, proprotein convertase subtilisin/kexin type 9; HMGCR, 3-hydroxy-3-methylglutaryl coenzyme A; NPC1L1, Niemann–Pick C1-Like 1; SNP, single-nucleotide polymorphism

**Fig. 2:**
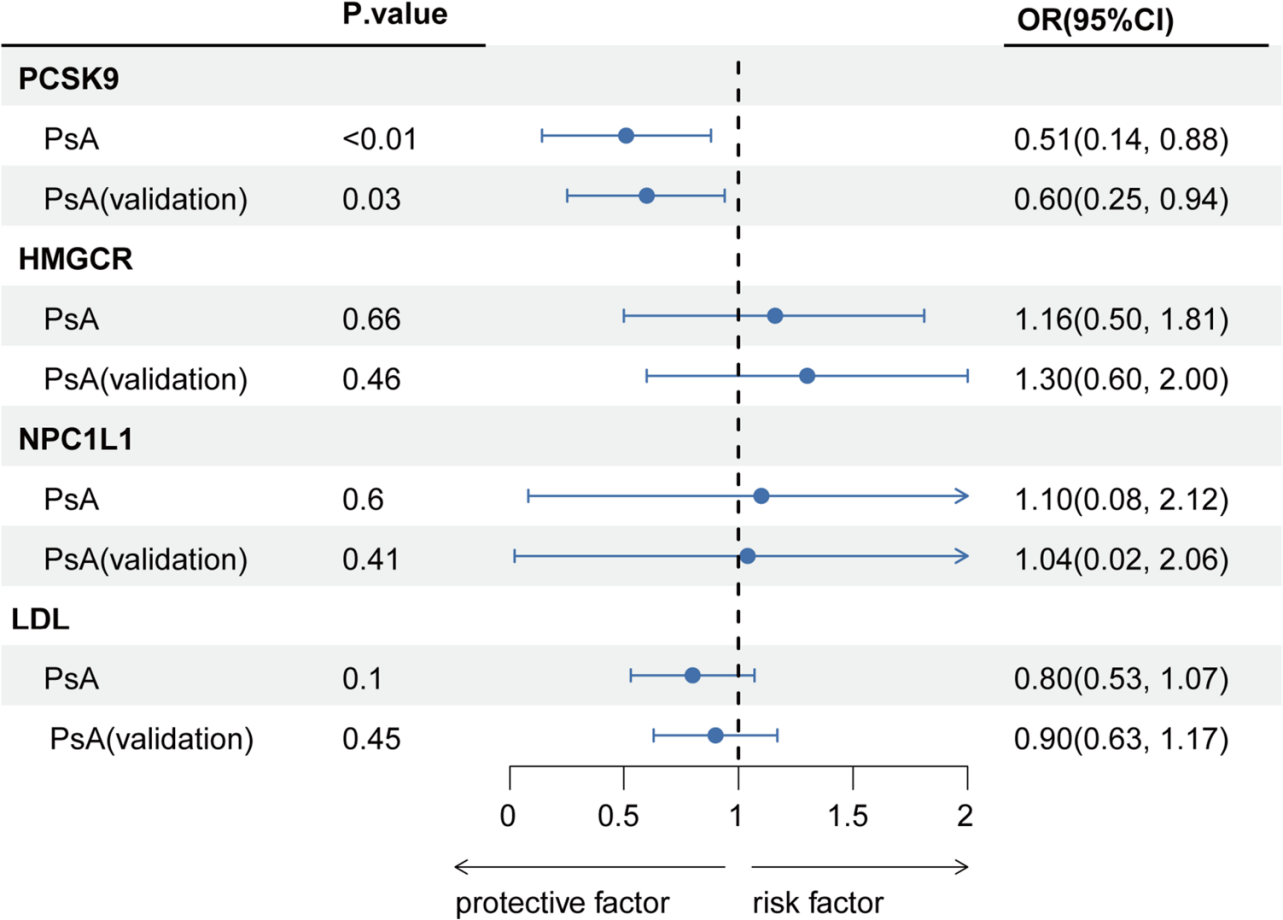
Associations between genetically proxied lipid-lowering drugs and psoriatic arthritis risk. PCSK9, proprotein convertase subtilisin/kexin type 9; HMGCR, 3-hydroxy-3-methylglutaryl CoA reductase; NPC1L1, Niemann–Pick C1-like 1; LDL, low-density lipoprotein; OR, odds ratio; PsA, psoriatic arthritis

**Fig. 3:**
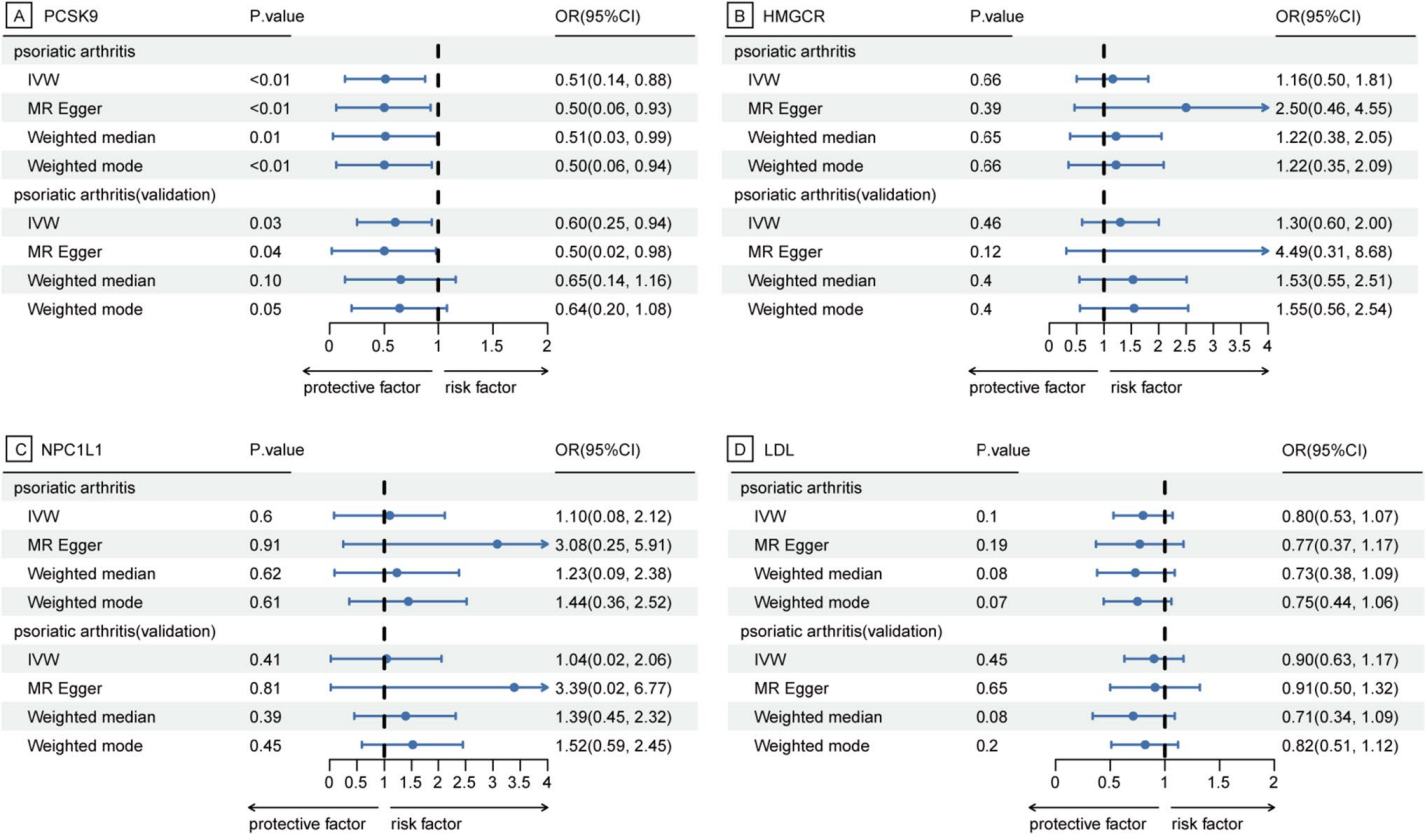
Summary of psoriatic arthritis risk results from pleiotropy robust sensitivity analyses. PCSK9, proprotein convertase subtilisin/kexin type 9; HMGCR, 3-hydroxy-3-methylglutaryl CoA reductase; NPC1L1, Niemann–Pick C1-like 1; LDL, low-density lipoprotein; IVW, inverse variance-weighted method; OR, odds ratio

**Fig. 4:**
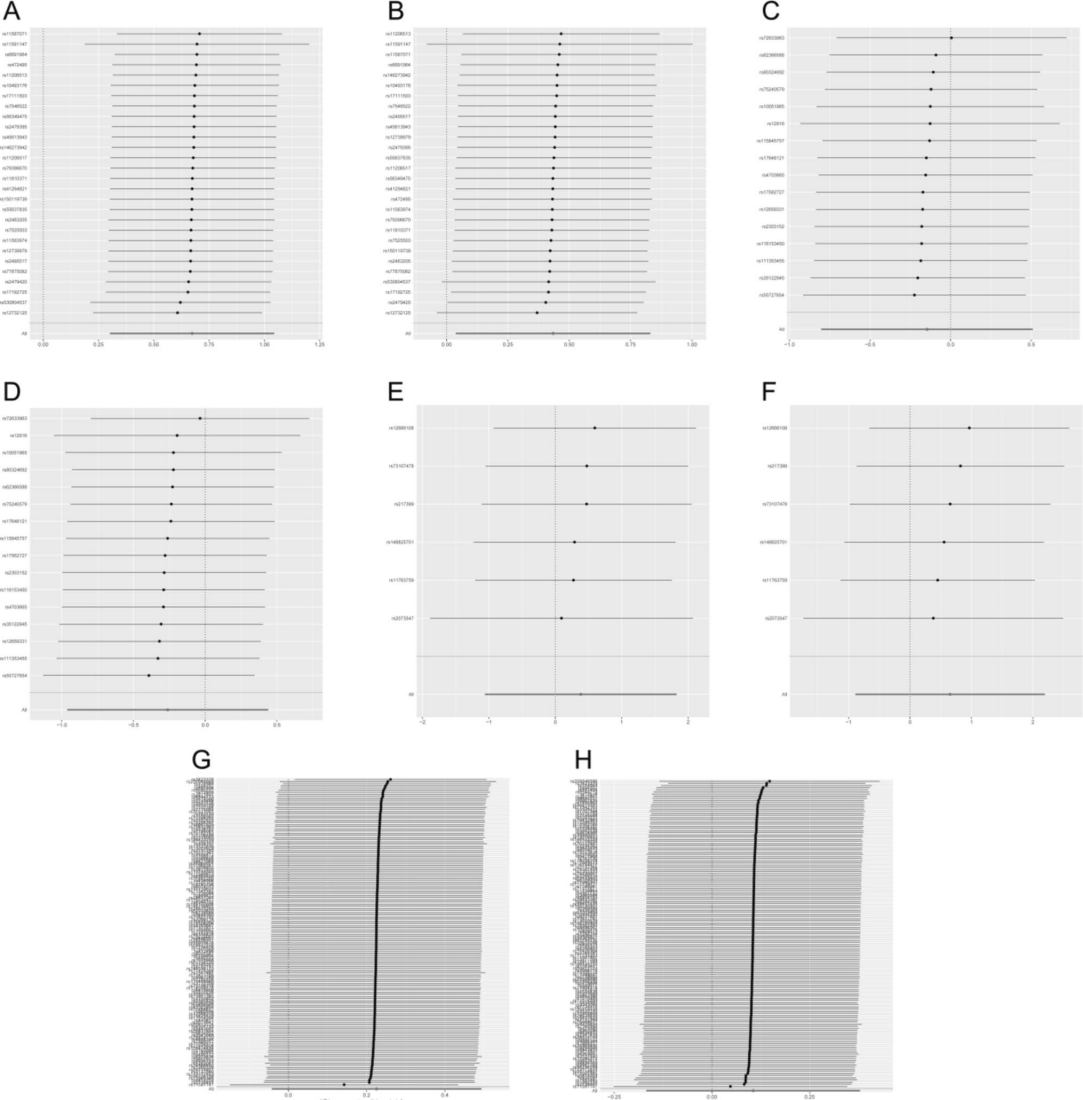
Sensitivity analysis of lipid-lowering drugs and LDL on psoriatic arthritis. Leave-one-out analysis of (**A)** PCSK9 on PsA, (**B)** PCSK9 on PsA (validation), (**C)** HMGCR on PsA, (**D)** HMGCR on PsA (validation), (**E**) NPC1L1 on PsA, (**F**) NPC1L1 on PsA (validation), (**G**) LDL on PsA, (**H**) LDL on PsA (validation). PCSK9, proprotein convertase subtilisin/kexin type 9; HMGCR, 3-hydroxy-3-methylglutaryl CoA reductase; NPC1L1, Niemann–Pick C1-like 1; LDL, low-density lipoprotein; PsA, psoriatic arthritis

Our findings indicate that genetically proxied PCSK9 inhibition correlates with a decreased PsA risk (odds ratio [OR]: 0.51; 95% CI 0.14–0.88; *P* < 0.01). This association was validated using an alternative GWAS dataset (OR 0.60; 95% CI 0.25–0.94; *P* = 0.03). There was no statistical heterogeneity in both the primary (*P* = 0.97) and validation estimates (*P* = 0.93), as depicted in Fig. 2, 3. Our sensitivity analyses yielded congruent results, with no discernible bias from horizontal pleiotropy, as illustrated in Fig. 3, 4 and eTable 2, 3.

Conversely, there was minimal evidence linking HMGCR with PsA risk across datasets (Figs. 2 and 3). The results for NPC1L1 mirrored this observation. Furthermore, the genetically inferred reduction in LDL, instrumented by 177 genetic variants (mean F statistic of 166) (eTable 4), showed no association with PsA in both primary and validation analyses.

**Fig. 5:**
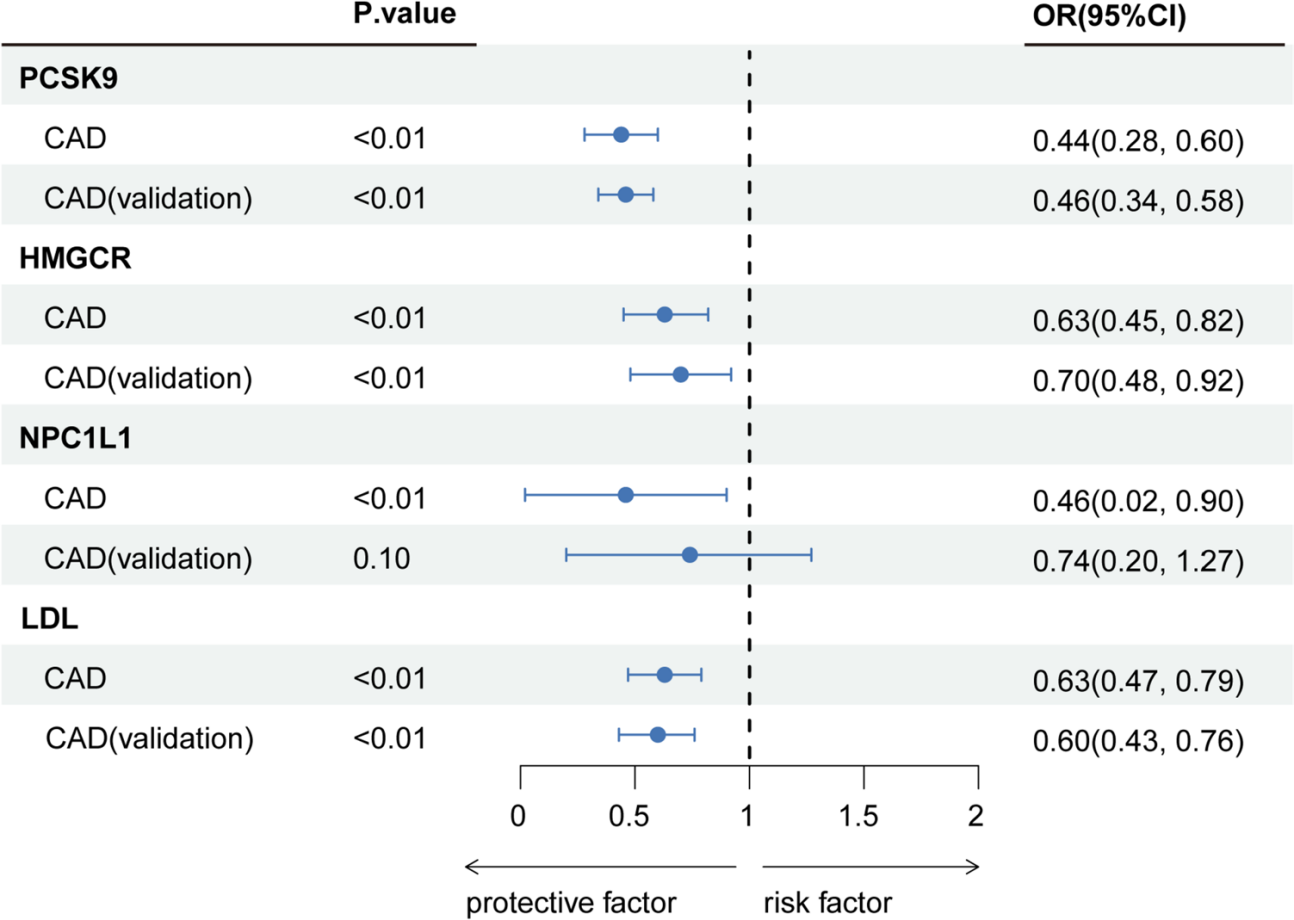
Correlations between genetically inferred lipid-lowering medications and coronary artery disease risk—positive control analysis. PCSK9, proprotein convertase subtilisin/kexin type 9; HMGCR, 3-hydroxy-3-methylglutaryl CoA reductase; NPC1L1, Niemann-Pick C1-like 1; LDL, low-density lipoprotein; CAD, coronary artery disease; OR, odds ratio

The genetic proxy for the inhibition of all three drug targets, as well as LDL, consistently demonstrated an association with a decreased risk of the positive control outcome, coronary artery disease, as illustrated in Fig. 5 and eTable 5. This association was evident in both the primary and validation analyses.

## Discussion

In this large-scale MR analysis, we delved into the impacts of three prevalent LDL-reducing drug targets (PCSK9 inhibitors, HMGCR—the target of statins, and NPC1L1 inhibitors—the target of ezetimibe) on the risks of PsA. Our findings underscore a causal link between PCSK9 inhibition and a diminished PsA risk, a relationship that seems independent of circulating LDL concentrations, as no overarching LDL–PsA risk association was discerned. Notably, genetic proxies for HMGCR and NPC1L1 inhibition showed no causal relationship with PsA risk.

PCSK9, a post-translational modulator of the LDL receptor (LDLR), orchestrates LDLR internalization and subsequent degradation. Its significance as a pivotal therapeutic target for hypercholesterolemia and coronary heart disease attenuation is evident [26]. A clinical study showed that the levels of PCSK9 in serum were moderately to severely correlated with the levels of LDL and total cholesterol (TChol) [27]. The FOURIER study demonstrated that PCSK9 inhibitors profoundly expunge LDL cholesterol from the circulatory system, leading to attenuated cardiovascular adversities [28].

While PCSK9's lipid-regulating function is universally acknowledged, its multifaceted functions extend to inflammation, gastrointestinal diseases, and viral infections [29–31]. Its role in inflammation, albeit pivotal, has often been relegated to the background. Research has pinpointed heightened PCSK9 expression elevating inflammatory chemokine production, including interleukin-1α, interleukin-6, and tumor necrosis factor-α (TNF-α) [32]. PCSK9 modulates the expression of TNF receptor-associated factors via the nuclear factor kappa-light-chain enhancer of activated B cells(NF-κB) signaling pathway [33, 34]. Moreover, PCSK9 is intertwined with several inflammatory pathways, encompassing the janus kinase/signal transducer and activator of transcription (JAK/STAT) pathway, TNF-α, and resistin [35, 36].

PCSK9 not only plays a crucial role in the regulation of serum LDL levels, but its biological function seems to extend beyond the regulation of cholesterol metabolism [37]. Current research indicates that PCSK9 is a key regulatory factor in the inflammation of chronic and autoimmune diseases [38]. In certain chronic conditions, such as chronic kidney disease and hypothyroidism, the expression and release of PCSK9 are also increased during the inflammatory process [39, 40].

Although the exact etiology of PsA remains elusive, prevailing research underscores the pivotal role of immune and inflammatory factors in its pathogenesis [3, 41]. PCSK9-mediated inflammatory cascades might be instrumental in the pathophysiological orchestration of PsA. Elevated serum PCSK9 concentrations have been documented in PsA cohorts [42]. PCSK9's potential to galvanize macrophage activation is noteworthy [43, 44]. Such macrophage activation, a hallmark of PsA pathogenesis, instigates a cascade of cytokines, notably Interleukin-17(IL-17), IL-1, TNF-α, and IL-23, which incite joint and entheseal inflammation, leading to cartilaginous and osseous degradation [45–47]. IL-17, pivotal in the inflammatory cascade, tissue damage, and bone erosion linked to PsA, has been spotlighted [48]. Elevated IL-17 levels and type 3 innate lymphoid cells, IL-17 producers, have been identified in PsA patient synovial fluid [49]. In hyperlipidemic mouse models, PCSK9 knockout reduced circulating IL-17 levels and the differentiation of IL-17-producing cells [50]. TNFα and IL1-β, pivotal inflammatory mediators in PsA, are suppressed in PCSK9-inhibited macrophages post-lipid exposure [51]. In conclusion, the data presented elucidates a significant association between PCSK9 and the pathophysiology of PsA. Coupled with our study outcomes, PCSK9 emerges as a promising therapeutic target.

Statins, ubiquitously employed lipid-lowering agents, are also prevalent among PsA patients to mitigate cardiovascular risk [52, 53]. Previous observational studies have probed the ramifications of statins on PsA's clinical trajectory, albeit with inconsistent outcomes [54–56]. Our study offers a resolution to this conundrum, revealing no causal ties between HMCGR inhibition and PsA susceptibility.

NPC1L1, a cholesterol uptake transporter protein predominantly expressed in the small intestine and liver. NPC1L1 inhibitor is another widely cited lipid-lowering agent [57]. To our knowledge, no extant research has elucidated NPC1L1 inhibitors causal relationship with PsA. Our findings indicate that genetic proxies for NPC1L1 inhibition remain unassociated with PsA, underscoring the need for further clinical validation.

This study, to the best of our knowledge, pioneers the MR approach to discern the causal effects of lipid-lowering agents on PsA. The robustness of our results, validated through replication and sensitivity analyses, is a notable strength. Nonetheless, this study has some limitations. Primarily, the MR analysis is susceptible to biases stemming from potential deviations from standard instrumental variable assumptions. Yet, multiple sensitivity analyses within our study found no indications of such violations, reinforcing the integrity of our primary results. Additionally, our study cohort was exclusively of European descent, underscoring the need for subsequent research in diverse ethnic groups to enhance the external validity of our conclusions.

## Conclusions

Our study elucidates that genetically proxied PCSK9 inhibition is inversely associated with psoriatic arthritis susceptibility, underscoring the therapeutic potential of extant PCSK9 inhibitors. Furthermore, this research suggests the possibility of personalized lipid-lowering drug selections for those with a predisposition to psoriatic arthritis. To cement these findings, subsequent randomized controlled trials are imperative.

## Data Availability

The GWAS summary statistics data used in this MR study is available in OpenGWAS (https://gwas.mrcieu.ac.uk/).
